# Nutritional status and growth pattern in children with cerebral palsy: A retrospective study from Qatar

**DOI:** 10.5339/qmj.2025.79

**Published:** 2025-08-17

**Authors:** Murad Abdulhafid Salem, Eman H.A. Abuhassan, Naeema Hassan Abdulla Al Dahneem, Amel Mohamed Daw, Kamaruddeen Mannethodi

**Affiliations:** 1 Pediatric Rehabilitation Department, Qatar Rehabilitation Institute, Hamad Medical Corporation, Doha, Qatar; 2 Corporate Nursing, Hamad Medical Corporation, Doha *Email: msalem11@hamad.qa

**Keywords:** Cerebral palsy, child growth and development, growth pattern, nutrition, nutritional status, pediatric nutrition

## Abstract

**Background:**

Children with cerebral palsy (CP) are at high risk of malnutrition due to feeding difficulties and motor impairments, which can further exacerbate their condition. Nutritional status plays a critical role in the growth and health outcomes of these children, yet limited data are available on this issue in Qatar. This study aimed to assess the nutritional status and growth patterns in children with CP attending a pediatric rehabilitation clinic in Qatar.

**Methods:**

A retrospective observational study was conducted on 150 children with CP, aged 3 to 14 years, who were followed regularly at the Pediatric Rehabilitation Department, Qatar Rehabilitation Institute. Nutritional status was evaluated using anthropometric measurements (height, weight, and body mass index [BMI]) based on World Health Organization growth charts. *χ*^2^ tests were performed to assess associations between BMI and demographic variables, including gender, nationality, motor type, weight, and height.

**Results:**

The prevalence of underweight children was 48.7% and 35.3% had an underweight BMI. Significant associations were found between BMI and nationality (*p* = 0.05), weight status (*p* < 0.001), and height status (*p* = 0.00(6). However, no significant associations were observed between BMI and gender (*p* = 0.21) or motor type (*p* = 0.2). Short stature and low weight were identified as strong indicators of undernutrition.

**Conclusions:**

Undernutrition remains a major concern among children with CP in Qatar, particularly among those with short stature and low weight. Tailored nutritional interventions and regular monitoring are essential to improve growth outcomes and prevent malnutrition-related complications in this vulnerable population.

## INTRODUCTION

Children affected with cerebral palsy (CP) often face malnutrition due to their limited motor or physical abilities, which may contribute to further deterioration of their condition.^1^ Understanding the nutritional status of children with CP is crucial for developing effective interventions and improving health outcomes in this vulnerable population. CP prevalence varies globally, with estimates ranging from 1.5 to 1.6 per 1,000 live births in high-income countries and as high as 3.4 per 1,000 live births in some low- and middle-income regions.^2^ In Arabic-speaking countries, the prevalence is reported at 1.8 per 1,000 live births, highlighting regional differences and the need for further research into CP epidemiology.^3^

CP is defined as a group of permanent disorders related to movement and posture development, hence causing activity limitations, which is attributed to nonprogressive disturbances that occurred during fetal or infant development of the brain.^4^ It is mainly characterized by motor function disorders, but often includes other dysfunctions entailing sensation, perceptual, cognitive, communication, behavioral, epilepsy, and secondary musculoskeletal disorders.^4,5^

In children, malnutrition can have a significant impact on their health and growth, as this is a period of rapid development. Malnutrition is a term used to describe either deficiencies, excesses, or imbalances in the intake of energy and nutrients. It entails two groups: undernutrition (stunting, wasting, underweight, micronutrient deficiencies) and overnutrition (overweight, obesity, diet-related noncommunicable diseases).^6^ Children with CP are often presented with feeding difficulties because of their motor disorders, which increases their risk of growth failure due to the resulting malnutrition from inadequate intake.^7^ A study in Norway reported reduced growth in weight and height throughout early childhood in those children who had feeding difficulties as an infant, compared to those who did not.^8^ Another study in Saudi Arabia found that more than half of the children with CP were malnourished and had slower growth.^9^ Furthermore, nutritional assessment is often difficult as the weight, height, growth, and body composition are different from a normal healthy developing child.^10^ All of this can translate into complications in adulthood, such as lung disease, musculoskeletal problems, mental health problems, as well as early death due to immune dysfunction and immobility.^11^ According to the World Health Organization (WHO), the relative risk of death in childhood is increased by 2.2× for moderate malnutrition and 6.8× for severe malnutrition.^12^ For children who are affected by CP, there are several factors other than motor dysfunction that can contribute to malnutrition. These include gastroesophageal reflux, delay in gastric emptying, poor posture, malabsorption, gastrointestinal problems, increased caloric demands, altered nutrient use, and nutrient provision limits due to fluid status and feeding tolerance, protein calorie malnutrition which impairs the cellular immune system and increases risk of infection, decreased response to vaccinations, as well as micronutrient deficiencies which lead to fatigue and muscle pain.^12^

Furthermore, nutrition status can be more significant in those children with increased severity of CP, as highlighted by one study, which showed that the prevalence of undernourishment increased with the increase of level in the Gross Motor Function Classification System (GMFCS), a tool used to categorize CP severity. Additionally, children with CP often exhibit growth patterns that deviate significantly from normal developmental trajectories, particularly in Arabic-speaking countries where the burden of malnutrition remains high.^3^

This study is the first of its kind in Qatar to comprehensively assess the nutritional status of children with CP. It provides valuable insights into the factors contributing to malnutrition within this population, including the unique interplay of motor impairments, feeding difficulties, and diverse socioeconomic and cultural influences. Qatar’s demographic diversity, encompassing various nationalities and socioeconomic backgrounds, offers a rich context for examining undernutrition in children with CP. By focusing on this multifaceted population, this research aims to fill critical gaps in knowledge and contribute to a broader understanding of CP-related malnutrition in the region. The primary objective of this study is to describe the nutritional status of children with CP attending the pediatric rehabilitation department at Qatar Rehabilitation Institute (QRI). A secondary objective is to explore the relationship between the degree of nutritional status and the type of CP, classified according to topographical categories. These findings will offer a foundation for future research and the development of targeted interventions aimed at improving the health outcomes of children with CP in Qatar.

## METHODS

### Study design

This study is a retrospective observational analysis conducted on children with CP attending the Pediatric Rehabilitation Nutritional Clinic at the QRI. Data were collected from 150 children, aged 3 to 14 years, who were followed up regularly between January 1, 2019, and January 1, 2022. The objective was to assess their nutritional status based on anthropometric measurements.

### Study population

The study included a total of 150 children diagnosed with CP. These patients were regularly followed up in the Pediatric Rehabilitation Nutritional Clinic, part of the QRI. These children met the inclusion criteria and had complete medical records and relevant anthropometric data available during the data collection period (January 2019 to January 2022). All eligible cases within the specified time frame were included in the analysis. No formal sample size calculation was performed, as this study utilized all available eligible cases within the specified time frame.

### Inclusion criteria

The study included patients with a confirmed diagnosis of CP, aged 3 to 14 years, during the data collection period. Only those who were regularly followed up at the pediatric rehabilitation dietitian clinic were included.

### Exclusion criteria

The study excluded children without CP (i.e., those with normal neurodevelopmental status), patients with neurological deficits unrelated to CP, syndromic patients, and patients diagnosed with CP but not regularly followed up at the pediatric rehabilitation dietitian clinic.

### Data collection

Data were retrospectively retrieved from the electronic medical record system (Cerner) of the Pediatric Rehabilitation Department, QRI. The dataset includes demographic and clinical characteristics, along with anthropometric measurements such as weight, height, and body mass index (BMI). The topographical classification of CP, describing the body parts affected (monoplegia, diplegia, triplegia, hemiplegia, quadriplegia), was also included for each case.

### Nutritional status assessment

Nutritional status was assessed based on anthropometric measurements. Weight-for-age, height-for-age, and BMI were evaluated using WHO growth charts, which are the approved standards for measuring children’s growth in Qatar. Underweight was defined as a BMI-for-age Z-score below –2 standard deviations (SD), normal weight as a BMI-for-age Z-score between –2 SD and +1 SD, and overweight as a BMI-for-age Z-score above + 1 SD.^13^ However, it is recognized that children with CP often exhibit different growth patterns compared to typically developing children. Some studies, such as Day et al., suggest the use of CP-specific growth references due to variations in growth trajectories.^14^ Despite this, WHO growth charts remain the most widely used and standardized tool for pediatric growth assessment in clinical and research settings. Findings from this study should be interpreted with consideration of these potential differences in growth patterns among children with CP.

### Study procedures

The classification of CP was based on the topographical distribution of motor impairment, specifically identifying the affected limbs.^15^ The topographical classifications used were monoplegia, diplegia, triplegia, hemiplegia, and quadriplegia. The study focused solely on the nutritional status of the participants as measured through height-for-age, weight-for-age, and BMI Z-scores. All measurements were compared to WHO growth standards to determine deviations from expected growth patterns.

### Ethical considerations

This study was conducted in full accordance with the principles outlined in the Declaration of Helsinki and adhered to the Good Clinical Practice guidelines, ensuring the ethical integrity of the research. All procedures followed the laws and regulations set forth by the Ministry of Public Health in Qatar. Ethical approval for this retrospective study was obtained from the Medical Research Center (MRC-01-22-708) at Hamad Medical Corporation. Since this is a retrospective study based on previously collected data, informed consent was not required from the participants.

### Statistical analysis

Descriptive statistics were used to summarize the demographic characteristics of the study participants, including gender, nationality, motor type, age range, weight status, height status, and BMI status. Frequencies and percentages were computed for categorical variables, providing an overview of the distribution of characteristics within the sample (*N* = 150).

The prevalence of different nutritional statuses (underweight, normal weight, and overweight) was calculated based on the number of children meeting each classification criterion (e.g., BMI- for-age *Z*-scores) relative to the total number of children included in the study (*N* = 150). For each category, the number of children classified as underweight, normal weight, or overweight was counted, and the prevalence percentage was derived by dividing the count of children in each group by the total study population of 150 children and multiplying by 100.

For inferential analysis, χ^2^ tests of independence were used to assess associations between categorical variables. The χ^2^ test was employed to evaluate the relationship between gender, nationality, motor type, age range, weight status, and BMI status.

All statistical analyses were performed using Statistical Package for Social Sciences (SPSS), version 23.0 (SPSS Inc., Chicago, IL)^16^ and Epiinfo software (Centers for Disease Control and Prevention, Atlanta, GA).^17^ The level of significance was set at p < 0.05.

## RESULTS

A total of 150 children with CP were included in the study. The analysis focused on their nutritional status, with data collected from the medical records through Cerner. Key findings regarding the prevalence of malnutrition and its association with the type and severity of CP are presented below. More than half of the subjects were boys (57%), and the BMI status shows 35% of the children were underweight. Children from 22 countries were included in the study, among whom the majority are Qatari nationals (34%) and Indians (15%). Most of the children belonged to the 6- to 10-year-old (48%). Additionally, they have varying motor abnormalities, such as diplegia (35.5%), hemiplegia (29.3%), quadriplegia (34%), ataxia (0.7%), and spastic paraplegia (0.7%). The detailed frequency distribution of demographic and population characteristic data is given in Supplementary Table 1.

Table 1 illustrates the association between gender and BMI status in the study population. The χ^2^ test results indicated no significant association between gender and BMI (*p* = 0.21). Both male and female participants were more likely to have a normal BMI, with 58.7% of boys classified as normal weight. Underweight status was observed in almost an equal share among boys and girls, while overweight status was more common among boys compared to girls. Despite these differences, the lack of statistical significance suggests that gender does not have a substantial impact on BMI distribution in this sample of children with CP.

The association between nationality and BMI status among children with CP revealed a significant association between nationality and BMI (*p* = 0.05). While the majority of children from most nationalities had a normal BMI, notable exceptions were observed. Specifically, 77% of Iranian children and all Pakistani children in the sample were classified as underweight. This suggests that certain nationalities may be more vulnerable to undernutrition, potentially due to socioeconomic or cultural factors that warrant further investigation. The details are given in Supplementary Table 2.

The association between different motor types and BMI status among children with CP is presented in Table 2. The χ^2^ test indicated no significant association between motor type and BMI (*p* = 0.2). Most children across different motor types had a normal BMI, particularly among those with diplegia (34.7%) and hemiplegia (34.7%). However, children with quadriplegia showed nearly equal proportions of normal (28.0%) and underweight (47.2%) BMI, suggesting a potential trend towards undernutrition in this group. Despite these trends, the lack of statistical significance indicates that motor type does not have a substantial influence on BMI status in this sample.

Table 3 presents the association between age group and BMI status in the study population, showing no significant difference between these two groups (*p* = 0.26). However, children in the 6- to 10-year-old group had the highest percentage (53.3%) of normal weight, and they tend to be more overweight (50.9%) compared to their younger and older counterparts. Contrastingly, higher prevalence of underweight and lower prevalence of overweight were shown in the 11- to 14-year-old age group. Despite these trends, the lack of statistical significance suggests that age does not have a strong influence on BMI status within the study sample.

Table 4 describes the association between weight status and BMI status. The statistical relationship shows a highly significant association between them (p ≤ 0.001). As expected, the majority (86.8%) of children classified as underweight also had an underweight BMI. Similarly, 69.3% of children with a normal weight status had a normal BMI. In contrast, all children classified as overweight by weight status fell within the overweight BMI category (54.5%). These findings confirm a strong relationship between weight status and BMI, with consistent categorization across both measures.

Table 5 demonstrates a highly significant association between height status with BMI status (*p* = 0.006). Most of the subjects with short height had fallen into the underweight (37.7%) or normal (25.3%) category of BMI status, with very few classified as overweight. In contrast, normal height subjects were evenly distributed across all three groups of BMI status. There is nobody in the underweight category among tall height participants.

## DISCUSSION

This is the initial investigation to document the nutritional status of infants with CP attending a pediatric rehabilitation clinic. The prevalence of malnutrition in our cohort, as defined by either indicator of nutritional status, is 35.3% lower in our data. The number of patients who are underweight in our study is lower than that of other studies conducted in the same region in Saudi Arabia, using the same WHO growth standards. The latter study determined that 56.4% of the patients were undernourished based on their weight for their age.^9^ This may be indicative of the effective health system we have implemented. Upon diagnosis of CP, all patients are immediately referred to the pediatric neurorehabilitation dietitian clinic, where they receive nutrition support before the onset of spasticity symptoms. Additionally, the patient is referred to the pediatric rehabilitation feeding and swallowing clinic for an assessment and the commencement of swallowing therapy by a multidisciplinary team.

In contrast, the prevalence of undernutrition in children with CP who attend rehabilitation centers in more affluent nations such as the USA, Argentina, and Greece is 52.9%, 38.1%, and a scant 7.9%, respectively.^18-20^ The notion that undernutrition is more prevalent in developing regions is substantiated by these disparities.^7^ These disparities may be attributed to the availability of more effective nutritional interventions in high-income countries, such as gastrostomy and comprehensive access to nutritionists and therapists who specialize in managing feeding difficulties in children with CP.^21^

In our study, we examined the relationship between gender and BMI status, but the statistical analysis showed no significant difference between the genders, even though a higher percentage of boys were overweight than girls. Both genders have more children with normal BMI, which indicates that gender is not an indicating factor for malnutrition. This finding is consistent with similar studies done among patients with CP, where gender does not determine the nutritional status.^22^ Due to the retrospective nature of data collection and reliance on existing medical records, alternative, more precise nutritional assessments could not be utilized, despite the recognized limitations of BMI in children with CP. Our study revealed a significant association between BMI status and nationality. Specifically, Iranian and Pakistani children had a higher prevalence of underweight BMI. These findings suggest that certain nationalities may face additional challenges related to socioeconomic factors and cultural practices. It is possible that these groups may experience barriers to adequate nutrition or face heightened risks due to differences in dietary habits and feeding support. This is a crucial finding that calls for targeted interventions that consider the socioeconomic and cultural contexts of different nationalities to reduce the risk of malnutrition. Although the study could not find any significant association between motor type and BMI status, children with quadriplegia showed a tendency towards underweight status, with nearly half of this group falling into the underweight group. Previous research done among children with neurological impairment, especially quadriplegics, suggests a greater risk for malnutrition.^23^ Our findings emphasize the need for specialized nutritional intervention for children with more severe motor impairment because they are at risk of malnutrition due to feeding difficulties, immobility, and increased caloric demands. In our research, we found that CP is associated with a low BMI in children as they grow.

The finding on the highly significant association of weight status and height status with BMI status reinforces the importance of these anthropometric measurements in assessing the nutritional status and confirming the validity of using BMI as a measure of nutritional status in this population. The study result showed that most underweight children by weight status classification also had an underweight BMI status, and those with normal weight status were most likely to have a normal BMI status. Similarly, short-stature children were most likely to be underweight or normal BMI, suggesting the link of height status to malnutrition. Also, children with normal height were evenly distributed across the BMI group, and interestingly, none of the tall children were underweight, and most had a normal or overweight BMI, reinforcing the link between growth and nutritional status. These findings highlight that short stature and low weight status are strong indicators of undernutrition, underscoring the need for targeted interventions to promote weight gain and growth in children with CP who are at higher risk for malnutrition.

Malnutrition in children with CP is associated with several complications, including increased risk of pneumonia, gastroesophageal reflux disease, osteopenia, fractures, contractures, and reduced immune function.^24^ While our study did not directly assess these complications, previous studies have demonstrated a strong link between undernutrition and these adverse outcomes. For example, studies have shown that underweight children with CP are more likely to develop aspiration pneumonia due to poor swallowing coordination, which can further exacerbate malnutrition.^25^ Additionally, inadequate calcium and vitamin D intake in malnourished patients with CP is associated with low bone mineral density, increasing the risk of fractures and skeletal deformities.^26^ Future studies in Qatar should investigate these complications in children with CP to guide targeted nutritional interventions and medical management.

Although children with CP aged 6 to 10 years appeared to have a higher proportion of normal or overweight BMI compared to other age groups, this difference was not statistically significant. However, potential factors such as variations in nutritional support, physical activity levels, and metabolic demands may contribute to age-related differences in BMI, warranting further investigation in larger cohorts. The proportion of patients with CP aged 11 to 14 years who were underweight was significantly higher than that of those with normal weight. The same discovery was made in a study conducted in Uganda, which demonstrated that children between the ages of 2 and 5 years are more susceptible to undernourishment than their younger counterparts under the age of 2 years. This indicates that the severity of underweight will increase as the child grows.^24^ In addition, we discovered that there is no correlation between the motor type of CP and nutritional status. However, there is a significant relationship between undernutrition and nationality. This suggests that the primary cause of undernutrition in CP is socioeconomic class. Therefore, additional research is necessary to investigate the underlying causes of malnutrition in CP.

The relationship between CP severity and nutritional status has been well-documented, with increasing motor impairment linked to higher rates of undernutrition. Studies suggest that children with more severe CP, particularly those classified at higher levels of the GMFCS, are at a greater risk of malnutrition due to feeding difficulties, reduced mobility, and increased energy expenditure.^27^ Similarly, topographical classifications (e.g., quadriplegia vs. hemiplegia) may have different impacts on growth patterns, as children with quadriplegia tend to have more severe feeding difficulties and lower weight-for-age compared to those with hemiplegia or diplegia. While this study did not directly assess CP severity using GMFCS or specific etiologies, our findings suggest that children with CP require individualized nutritional monitoring based on their motor impairment and functional status.

Swallowing dysfunction and the need for enteral feeding are additional key factors affecting growth in children with CP. Studies have shown that oropharyngeal dysphagia is prevalent in children with CP and significantly impacts caloric intake, leading to growth failure. The presence of a feeding tube (nasogastric or gastrostomy) can influence nutritional outcomes by ensuring adequate nutrient intake, but it may also introduce challenges in weight and growth monitoring.^28^ Future studies should explore the impact of swallowing difficulties and feeding tube use in children with CP in Qatar to better tailor nutritional interventions.

## LIMITATIONS

A key limitation of this study is the retrospective nature of the study design, which relied on medical record data, potentially introducing information bias or missing data by excluding patients who may have incomplete records or are not consistently followed up. Additionally, the study includes children from various nationalities; the small sample size of certain groups may limit the generalizability of the study. Future research with a larger and more diverse sample with a prospective study design could provide deeper insights into the factors contributing to malnutrition in this population.

## CONCLUSIONS

Malnutrition is prevalent among children with CP in Qatar’s Pediatric Rehabilitation Department, with 35.3% classified as underweight based on BMI. Children aged 5 years and older were found to be at higher risk of malnutrition, highlighting the need for targeted nutritional interventions. Efforts should focus on implementing tailored nutritional care plans, regular growth monitoring, and individualized management strategies to improve nutritional outcomes and support the healthy development of children with CP.

Challenges in assessing nutritional status in this population include feeding difficulties, the lack of standardized growth references specific to children with CP, and variations in socio-demographic factors. Addressing these barriers will require coordinated efforts by the Health Ministry and key stakeholders, including the establishment of more government-supported rehabilitation institutions for children with special needs.

Additionally, future research should focus on conducting multicenter, prospective studies to examine the factors contributing to malnutrition, such as caloric intake, feeding challenges, and micronutrient deficiencies. Such studies could also evaluate the long-term impact of targeted interventions and explore region-specific solutions for improving the nutritional status of children with CP in Qatar. These findings would contribute to evidence-based guidelines for optimizing nutritional care and mitigating malnutrition-related complications in this vulnerable population.

### Authors’ contribution

MAS contributed to the concept, study design, and manuscript writing. EHAA and NHAAD were responsible for data collection, data management and provided technical advice. AMD contributed to the review and editing of the manuscript. KM was involved in manuscript writing and review. All authors have read and approved the final version of the manuscript.

### Acknowledgements

The authors would like to express their sincere gratitude to Dr. Prem Chandra, Academic Research Scientist, Hamad Medical Corporation, for their valuable contributions to the statistical analysis of this study. Also, the authors would like to acknowledge Dr. Mahmoud Ibrahim Abeidah, Head of Pediatric Rehabilitation, and Dr Hanadi Khamis,Medical Director, QRI, Hamad Medical Corporation, for their valuable support and guidance throughout the course of this study.

### List of abbreviations

**Table:** 

BMI	Body Mass Index
CP	Cerebral Palsy
GMFCS	Gross Motor Function Classification System
QRI	Qatar Rehabilitation Institute
SD	Standard Deviation
SPSS	Statistical Package for Social Sciences
WHO	World Health Organization

### Funding

No funding was received for this study.

### Conflict of interest

The authors declare that they have no conflicts of interest to disclose related to this study.

## Figures and Tables

**Table 1.** Association between gender and body mass index (BMI) status by χ^2^.

			BMI status			
			Underweight	Normal	Overweight	Total
**Gender**	**Boys**	Count	27	44	16	87
		% within BMI status	50.9%	58.7%	72.7%	58.0%
	**Girls**	Count	26	31	6	63
		% within BMI status	49.1%	41.3%	27.3%	42.0%
	**Total**	Count	53	75	22	150

**Table 2.** Association between motor type and body mass index (BMI) status.

			BMI status			
			Underweight	Normal	Overweight	Total
Motor type	Diplegia	Count	18	26	9	53
		% within BMI status	34.0%	34.7%	40.9%	35.3%
	Hemiplegia	Count	10	26	8	44
		% within BMI status	18.9%	34.7%	36.4%	29.3%
	Quadriplegia	Count	25	21	5	51
		% within BMI status	47.2%	28.0%	22.7%	34.0%
	Ataxia	Count	0	1	0	1
		% within BMI status	0.0%	1.3%	0.0%	0.7%
	Spastic paraplegia	Count	0	1	0	1
		% within BMI status	0.0%	1.3%	0.0%	0.7%
Total			53	75	22	150

**Table 3.** Age range and body mass index (BMI) status association.

			BMI status			
			Underweight	Normal	Overweight	Total
Age range	3 to 5 years old	Count	17	20	9	46
		% within BMI status	32.1%	26.7%	40.9%	30.7%
	6 to 10 years old	Count	21	40	11	72
		% within BMI status	39.6%	53.3%	50.0%	48.0%
	11 to 14 years old	Count	15	15	2	32
		% within BMI status	28.3%	20.0%	9.1%	21.3%
Total		Count	53	75	22	150

**Table 4.** Association between weight status and body mass index (BMI) status.

			BMI status			
			Underweight	Normal	Overweight	Total
Weight status	Under weight	Count	46	23	4	73
		% within BMI status	86.8%	30.7%	18.2%	48.7%
	Normal	Count	7	52	6	65
		% within BMI status	13.2%	69.3%	27.3%	43.3%
	Overweight	Count	0	0	12	12
		% within BMI status	0.0%	0.0%	54.5%	8.0%
Total		Count	53	75	22	150

**Table 5.** Association between height status and body mass index (BMI) status.

			BMI status			
			Underweight	Normal	Overweight	Total
Height status	Short	Count	20	19	1	40
		% within BMI status	37.7%	25.3%	4.5%	26.7%
	Normal	Count	33	55	19	107
		% within BMI status	62.3%	73.3%	86.4%	71.3%
	Tall	Count	0	1	2	3
		% within BMI status	0.0%	1.3%	9.1%	2.0%
Total		Count	53	75	22	150

## Supplementary tables

Supplementary Tables S1-S2
