# Supplementary material for: Nutritional status and growth pattern in children with cerebral palsy: A retrospective study from Qatar
Source: Qatar Med J. 2025 Aug 17;2025(3):79. doi: 10.5339/qmj.2025.79 (PMC12893714; doi:10.5339/qmj.2025.79)
Supplement: Supplementary Tables S1-S2 [file qmj.2025.79supplement.pdf]

Supplementary tables

Supplementary table 1: Descriptive statistics of Demographic Variables (N = 150)

Characteristics	Frequency	Percentage
**Gender**		
Male	87	58
Female	63	42
**Nationality**		
Qatari	52	34.7
Indian	23	15.3
Egyptian	14	9.3
Jordanian	5	3.3
Sudanese	10	6.7
Syrian	9	6.0
British	3	2.0
Bahraini	2	1.3
American	1	0.7
Iranian	3	2.0
Sri Lankan	1	0.7
Pakistani	9	6.0
Tunisian	3	2.0
Yemeni	4	2.7
Saudi	1	0.7
Algerian	2	1.3
Lebanese	1	0.7
Bangladeshi	2	1.3
Filipino	1	0.7
Afghan	1	0.7
Iraq	1	0.7
Canadian	1	0.7
**Motor Type**		
Diplegia	53	35.5
Hemiplegia	44	29.3
Quadriplegia	51	34.0
Ataxic	1	0.7
Spastic paraplegia	1	0.7
**Age range**		
3 to 5 years old	46	30.7
6 to 10 years old	72	48.0
11 to 14 years old	32	21.3
**Weight status**		
Under weight	73	48.7

Normal	65	43.3
Overweight	12	8
**Height status**		
Short	40	26.7
Normal	107	71.3
Tall	3	2
**BMI status**		
Under weight	53	35.3
Normal	75	50.0
Overweight	22	14.7

Supplementary table 2: Nationality and Association with BMI

			BMI status			
			Underweight	Normal	Overweight	Total
Nationality	Qatari	Count	14	29	9	52
		% within BMI status	26.4%	38.7%	40.9%	34.7%
	Indian	Count	10	11	2	23
		% within BMI status	18.9%	14.7%	9.1%	15.3%
	Egyptian	Count	5	2	7	14
		% within BMI status	9.4%	2.7%	31.8%	9.3%
	Jordanian	Count	1	3	1	5
		% within BMI status	1.9%	4.0%	4.5%	3.3%
	Sudanese	Count	3	7	0	10
		% within BMI status	5.7%	9.3%	0.0%	6.7%
	Syrian	Count	4	5	0	9
		% within BMI status	7.5%	6.7%	0.0%	6.0%
	British	Count	0	2	1	3
		% within BMI status	0.0%	2.7%	4.5%	2.0%
	Bahraini	Count	1	1	0	2
		% within BMI status	1.9%	1.3%	0.0%	1.3%
	American	Count	1	0	0	1
		% within BMI status	1.9%	0.0%	0.0%	0.7%
	Iranian	Count	3	0	0	3

	% within BMI status	5.7%	0.0%	0.0%	2.0%
Sri Lankan	Count	0	1	0	1
	% within BMI status	0.0%	1.3%	0.0%	0.7%
Pakistani	Count	7	2	0	9
	% within BMI status	13.2%	2.7%	0.0%	6.0%
Tunisian	Count	1	1	1	3
	% within BMI status	1.9%	1.3%	4.5%	2.0%
Yemeni	Count	1	3	0	4
	% within BMI status	1.9%	4.0%	0.0%	2.7%
Saudi	Count	0	0	1	1
	% within BMI status	0.0%	0.0%	4.5%	0.7%
Algerian	Count	0	2	0	2
	% within BMI status	0.0%	2.7%	0.0%	1.3%
Lebanese	Count	1	0	0	1
	% within BMI status	1.9%	0.0%	0.0%	0.7%
Bangladeshi	Count	0	2	0	2
	% within BMI status	0.0%	2.7%	0.0%	1.3%
Filipino	Count	0	1	0	1
	% within BMI status	0.0%	1.3%	0.0%	0.7%
Afghan	Count	0	1	0	1
	% within BMI status	0.0%	1.3%	0.0%	0.7%
Iraq	Count	0	1	0	1
	% within BMI status	0.0%	1.3%	0.0%	0.7%
Canadian	Count	0	1	0	1
	% within BMI status	0.0%	1.3%	0.0%	0.7%
Total	Count	52	75	22	150
